# The Knowledge Translation Complexity Network (KTCN) Model: The Whole Is Greater Than the Sum of the Parts - A Response to Recent Commentaries

**DOI:** 10.15171/ijhpm.2018.49

**Published:** 2018-06-11

**Authors:** Alison Kitson, Rebekah O’Shea, Alan Brook, Gill Harvey, Zoe Jordan, Rhianon Marshall, David Wilson

**Affiliations:** ^1^College of Nursing and Health Sciences, Flinders University, Bedford Park, SA, Australia.; ^2^Green Templeton College, University of Oxford, Oxford, UK.; ^3^University of Adelaide, Adelaide, SA, Australia.; ^4^Adelaide Dental School, Faculty of Health and Medical Sciences, University of Adelaide, Adelaide, SA, Australia.; ^5^Institute of Dentistry, Queen Mary University of London, London, UK.; ^6^Adelaide Nursing School, Faculty of Health and Medical Sciences, University of Adelaide, Adelaide, SA, Australia.; ^7^Alliance Manchester Business School, University of Manchester, Manchester, UK.; ^8^The Joanna Briggs Institute, Faculty of Health and Medical Sciences, University of Adelaide, Adelaide, SA, Australia.; ^9^Independent Clinical Psychologist.; ^10^Adelaide Medical School, Faculty of Health and Medical Sciences, University of Adelaide, Adelaide, SA, Australia.

“*The mind once stretched by a new idea, never returns to its original dimensions.*”

Ralph Waldo Emerson

We would like to thank the authors who provided commentaries to our article. It is reassuring that our endeavours to explain a complex set of ideas and relationships are being carefully evaluated. Those who wrote commentaries supported to a greater or lesser degree the complexity inherent in knowledge translation (KT) as outlined in the model. We appreciated both confirmatory comments about the characteristics of the Knowledge Translation Complexity Network (KTCN) model and the observations that could lead to further refinement. This process aligns with the evaluation element in the model.

Sturmberg^1^ reminded us of the value in distinguishing between data, information, facts, knowledge and wisdom, and that organisations ‘don’t receive knowledge’; they have to ‘learn’ about it. Sturmburg adds the extent to which organisations learn new knowledge, may be related to their purpose, goals and values. As the KTCN model is refined we agree there will be value in including reference to the inherent complex nature of knowledge and organizational behaviours. Taking a more traditional knowledge management perspective, Carlile^2^ argued that there were three types of knowledge movement: knowledge transfer, translation and transformation, each depending on how the knowledge had to be altered or adapted into the setting. This notion is consistent with Sturmberg’s ideas but also acknowledges the interactivity between the knowledge and the context.

Chandler^3^ adds that more systematic work needs to be done on understanding how complexity theory can be used in health care settings. She also noted the value in having consistency in nomenclature, and greater alignment in order to enhance progress and deeper understanding. A major point of debate was the inherent paradox of whether the proposed KTCN model was purported to be predictive as well as explanatory. To clarify, retrospective mapping using the KTCN model can be used to explain behavior.^4,5^ The model is not intended to be predictive, but rather is a tool to be used to work a solution to a problem. The very nature of complexity and the need for evaluation at each step often means the problem, and solution, may change. As such, we agree that claiming to control or change behaviour is the anathema of how complexity thinking works. Indeed, the notion of ‘control’ is inappropriate: the strength may not be in ‘making’ the system change; it may be in recognizing and working with it, rather than expecting something will result from pushing against it.

Rycroft-Malone^6^ noted that the utility of such a model will be contingent upon how easy users find it to understand and apply to their ‘challenge’ (or wicked problem), and to do so in a way that helps with both explanation and prediction. Perhaps understanding the emerging (and potentially different) solution is progress towards the solution. This addresses concerns of Kothari and Sibbald in relation to providing a solution.^7^

Kirchner et al^8^ provided a worked example of how the KTCN model can be used to explain the different actions and interactions between agents and actors within a complex implementation project. Using a multi-site implementation project introducing ways to reduce suicide rates in Veterans within Veterans Affairs, they demonstrated how using the model helped to make sense of what happened – both the expected and unintended consequences. They raised an important point regarding the negotiation of the timing and the execution of the project, taking into account the aims of multiple stakeholders, which in turn influence and shape the process and potential outcomes of the project.

Bucknall and Hitch^9^ recognized the complexity and unpredictability of KT and the need to examine complex adaptive systems from diverse perspectives. They refer to the KTCN model as descriptive and ‘coherent, and it enables comparison of the properties with other theories’ (p3). The model does not intend to explain all, as we recognize one prescriptive approach cannot fit all. Yet, it can add to the move away from linear, reductionist thinking to a more contingent, networked, relationship based approach. Their analysis of the KTCN model against Nilsen’s typology^10^ was very helpful, and a line of inquiry that could further support Chandler’s request for more systematic evaluation.

From these discrete reviews, we wish to reiterate a foundational point; namely that the KTCN model encourages us to change the way we think about how knowledge moves within and across systems. Our thesis in the paper was that the shift in thinking needs to happen in the first instance, within the individual, acknowledging that there is rarely a completely prescribed way of doing (KT) things. Raising the awareness of the complexity and dynamism of situations, the individual can learn to use the KTCN model scaffolding as an external aid rather than starting from the (more accepted) position that if a set of KT tools are generated and implemented, then desired outcomes will be achieved. This is the reason evaluation of complexity is both required and demanding. It may also be part of the reason why our understanding of the effectiveness of certain implementation interventions is equivocal – we are unable to recognize and hence describe the multiple variations and nuanced interactions that necessarily take place in order for desired goals to happen.

It may be that the first step is around understanding the nature and extent of the problem. Problems will have different ‘urgencies’ – as alluded to by Chandler’s comment (p2); tragedies (such as the Tsunami example in the paper) will galvanise concerted action, more so than contested issues. Tragedies, wars, power struggles and competitive advantage tend to create their own urgencies while other (equally important but contested) social issues such as fighting obesity or poverty or indeed, ensuring that health professionals use appropriate hand washing techniques, do not seem to have the same traction despite policy support and community involvement. This means that we enact KT by ‘pulling’ relevant knowledge through to groups and then the networks enable the appropriate connections to happen. The ‘pulling’ may indeed be a function of the concentrated energy and will to act, alongside the identification of the relevant champions – or actors – who can enable things to happen.

Coming together around trying to solve these complex problems is what is happening in the research world more and more. We know that universities globally are incentivizing trans-disciplinary and inter-organizational teams to work more effectively together. By engaging health professionals, corporates, government representatives and potential consumers from the start, it is anticipated that there will be greater readiness and receptivity to implementation and evaluation.

However, there are still multiple challenges if we embrace this way of thinking about KT.

The majority of the reviewers confirmed the elements of the model – dynamism, complexity, emergence, timelines – but wanted more recognition around how these elements fit and how they can be used. Players and sub-networks are different so again, how does this work and how is it applied? Expectations from some of the commentaries are that you can generate a tool and make KT happen, however this way of thinking does not align with the inherent flexibility that the model encourages. We believe it is important to embrace the KTCN model as a concept that offers a different way of thinking and is not simply a circularized linear model with complexity. We are coming back to the realization that all you can do is identify the elements and then launch yourself into the ‘workflow that is a natural experiment’ – it has never been done before, it will not ever happen in the same way again but the shared understanding, experience and knowledge will help individuals, teams and organisations ‘do better’ next time.

Again evaluation of processes is key and it does alert one to patterns that are both static and dynamic. Our current understanding of evaluation methods and processes often does not enable us to exercise this level of specificity or depth. Evaluation needs to be continuous, both at the individual and organizational level; it starts with reflective practice and builds up into a wider systems process with multiple actors/agents/actions involved. It is methodologically challenging but with data and feedback, it can start to provide agents in complex systems with approaches to understand how best to act in order to achieve their goal.

This leads us to consider how individual reflections can be validated by sharing and confirmation, refutation and pattern recognition. These are the boundaries where individual experiences can be aggregated into networks of shared understanding, leading to what Sturmberg would describe as wisdom. Does this reflect the nodes, hubs and networks in action?

So, what are the next steps in this journey? Perhaps the best way is to follow on from Kirchner et al,^8^ and do a post hoc analysis of a previous problem. This sets the mental processes in place enabling flexible navigation and application to a currently unresolved wicked problem.

## Acknowledgements

We would like to thank the considered feedback on the KTCN model as provided through the commentaries.

## Ethical issues

Not applicable.

## Competing interests

Gill Harvey is on the Editorial Board for the *International Journal of Health Policy and Management.*

## Authors’ contributions

AK and RO drafted the manuscript. All authors critically reviewed and revised the manuscript. All authors were involved with the development of the original article to which the commentary relates.

## Authors’ affiliations

^1^College of Nursing and Health Sciences, Flinders University, Bedford Park, SA, Australia. ^2^Green Templeton College, University of Oxford, Oxford, UK. ^3^University of Adelaide, Adelaide, SA, Australia. ^4^Adelaide Dental School, Faculty of Health and Medical Sciences, University of Adelaide, Adelaide, SA, Australia. ^5^Institute of Dentistry, Queen Mary University of London, London, UK. ^6^Adelaide Nursing School, Faculty of Health and Medical Sciences, University of Adelaide, Adelaide, SA, Australia. ^7^Alliance Manchester Business School, University of Manchester, Manchester, UK. ^8^The Joanna Briggs Institute, Faculty of Health and Medical Sciences, University of Adelaide, Adelaide, SA, Australia. ^9^Independent Clinical Psychologist. ^10^Adelaide Medical School, Faculty of Health and Medical Sciences, University of Adelaide, Adelaide, SA, Australia.
